# Risk factors for 1-year peripheral neuropathy and F-wave abnormalities after COVID-19: a prospective cohort study

**DOI:** 10.3389/fneur.2025.1532046

**Published:** 2025-07-08

**Authors:** Qian Yang, Yingying Chang, Yinuo Zhao, Yanhui Lai, Xuzhao Liu, Ying Xiao, Na Li, Shuhua Cui, Zhibo Wang, Ling Cui, Yubin Zhao

**Affiliations:** ^1^Haikou Fourth People’s Hospital, Haikou, Hainan, China; ^2^Hebei University of Chinese Medicine, Shijiazhuang, Hebei, China; ^3^School of Biological Sciences, Faculty of Biology, Medicine and Health, The University of Manchester, Manchester, United Kingdom; ^4^Shijiazhuang People’s Hospital, Shijiazhuang, Hebei, China; ^5^North China University of Science and Technology, Tangshan, Hebei, China; ^6^College of Chemical Engineering, Shijiazhuang University, Shijiazhuang, Hebei, China

**Keywords:** COVID-19, peripheral neuropathy, mononeuropathy, F-wave abnormality, risk factors

## Abstract

**Objective:**

To explore the risk factors for peripheral neuropathy (PNP) and F-wave abnormality during one-year rehabilitation for coronavirus disease 2019 (COVID-19).

**Methods:**

This prospective cohort study included patients with COVID-19 who were discharged from Shijiazhuang People’s Hospital after treatment and routine rehabilitation between December 2020 and April 2021. Multivariable logistic regression analysis was used to analyze the independent risk factors for PNP and F-wave abnormality. *p*-values <0.0125 were considered significant in the multivariable analyses to account for type I error.

**Results:**

A total of 313 patients with COVID-19 [aged 49.0 (IQR 33.0–58.0) years, and 191 (61.0%) females] were included. During one-year follow-up, 232 (74%) patients developed PNP (PNP group), and 81 (26%) did not (non-PNP group). In the PNP group, 51 (16%) patients had mononeuropathy, and 181 (58%) had polyneuropathy. Additionally, F-wave abnormality was detected in 22 (7%) out of 313 patients. Multivariable logistic regression analysis showed that age [odds ratio (OR) = 1.22, confidence interval (CI): 1.05–1.41, *p* = 0.009] was independently associated with PNP. College or higher education (OR = 5.07, 95% CI: 1.80–13.90, *p* = 0.002) was independently associated with mononeuropathy.

**Conclusion:**

Age might be an independent risk factor for PNP, while higher education was associated with mononeuropathy.

## Introduction

COVID-19 predominantly affects the lungs but can also harm the nervous, cardiovascular, and renal systems, especially in hospitalized patients (1–3). Still, recent studies reported localized pain and numbness in some COVID-19 patients, suggesting the involvement of the peripheral nervous system (2, 4, 5).

Peripheral neuropathy (PNP) refers to conditions that involve damage to the peripheral nervous system, resulting in sensory symptoms (e.g., numbness, tingling), weakness, autonomic symptoms (e.g., early satiety, sweat abnormalities), and neuropathic pain (6). Electrodiagnostic studies are valuable for patients with suspected PNP; electrodiagnostic testing includes nerve conduction studies (NCS) that can determine the existence of PNP and the scope of lesions and evaluate damaged parts and pathological type (7). In the case of a positive diagnosis, early nerve nutrition drugs are given in combination with rehabilitation treatment to avoid more serious physical symptoms (8).

A review of 105 studies demonstrated that PNP is prevalent in patients with COVID-19, primarily due to immune mechanisms or neurotoxic side effects of medications; few patients developed PNP from prolonged bedding in the intensive care unit (ICU) and pre-existing diabetes (9). Because of the severe damage to multiple organs caused by the novel coronavirus, many patients continue to experience a range of physical and psychological symptoms beyond the acute phase of COVID-19 infection, including PNP-related symptoms like pain, limb numbness, and muscle weakness (10, 11). Previous studies also showed that 60% of the patients have persistent pain and numbness even after treatments, and these prolonged symptoms can lead to anxiety, depression, reduced productivity, and even suicide, increasing the economic burden on the family and healthcare system (12).

Despite the long-term impact of PNP, most studies on PNP in COVID-19 patients are based on case reports (13, 14). Furthermore, the risk factors of PNP in COVID-19 patients have not been explored. A better understanding of the risk factors can help healthcare professionals to identify high-risk individuals and intervene early. Therefore, this study investigated the risk factors of PNP among COVID-19 survivors at 6-month and 12-month follow-ups after diagnosis using NCS.

## Materials and methods

### Study design and participants

This prospective cohort study included patients with COVID-19 who were discharged from Shijiazhuang People’s Hospital after treatment and routine rehabilitation between December 2020 and April 2021. All eligible, consecutive, and willing-to-participate patients were included. All the enrolled patients received rehabilitation treatment 2 to 4 weeks after discharge. Two follow-up visits were conducted 6 and 12 months after hospital discharge and performed in the COVID-19 rehabilitation clinic.

The inclusion criteria were (1) age 10–70 years old, (2) diagnosed and hospitalized with COVID-19 as the primary diagnosis, (3) discharged from the hospital after meeting the discharge criteria in the Eighth Edition of the Chinese Clinical Guidance for COVID-19 Pneumonia Diagnosis and Treatment (15), (4) received rehabilitation treatment for 2 to 4 weeks after discharge, and (5) completed nerve conduction velocity examinations including motor nerve conduction latency, amplitude and velocity/sensory nerve conduction latency, amplitude, and velocity. The exclusion criteria were (1) having pre-existing conditions that could lead to PNP, including immune system diseases, neuromuscular diseases, malignant tumors, diabetes, fractures, cervical spondylosis, or lumbar spondylosis, (2) those who were bedridden or with limited mobility, (3) with history of mental disorders, which is a contraindication for NCS, (4) those who were unable to cooperate with researchers, or (5) with incomplete medical record or tests.

This study was reviewed and approved by the institutional ethics committee of Shijiazhuang People’s Hospital, and all participants signed the informed consent form. This study was registered in clinicaltrials.gov (ChiCTR2100052291).

### Procedures

All the enrolled patients received rehabilitation treatment 2 to 4 weeks after discharge, including cardiopulmonary function recovery, psychological counseling, and traditional Chinese medicine treatment. Demographic data, type of treatment received, blood glucose levels at admission and discharge, and levels of novel coronavirus (nCoV) immunoglobulin G (IgG) at discharge were obtained from the electronic medical records. Comorbidity was reported by the patient and confirmed by medical records and test results (i.e., imaging and laboratory tests) during hospitalization. COVID-19-related symptoms at disease onset, including fever, cough, dyspnea, fatigue, nausea or vomiting, diarrhea, myalgia, chest tightness, and sputum production, were documented based on self-report. All participants were classified as mild, moderate, severe, and critically severe according to the Eighth Edition of the Chinese Clinical Guidance for COVID-19 Pneumonia Diagnosis and Treatment (15).

Two follow-up visits were conducted 6 and 12 months after hospital discharge and performed in the COVID-19 rehabilitation clinic. During the follow-up visits, patients underwent NCS using full-featured electromyography/evoked potential equipment; NCS included motor nerve conduction and sensory nerve conduction measurements (16). The measurements were completed by a senior physician with experience in neurophysiology, according to the standardized methods (17). The procedures and criteria are shown in the Supplementary file.

Before testing, it was verified that skin temperature was 30–32°C. Disk-shaped surface electrodes were used to record the compound muscle action potential (CMAP) and sensory nerve action potential (SNAP). When measuring motor conduction, the cathode was placed at the distal end, and the anode was placed at the proximal end. When the F-wave was measured, the cathode was placed at the proximal end. In retrograde sensory conduction measurement, the stimulating electrode was placed on the nerve stem, the cathode was at the distal end, and the anode was at the proximal end. The distance between the cathode and the anode was about 2 cm. When motor conduction was measured, the acting electrode was placed on the belly of the muscle, and the reference electrode was placed on the tendon or its attachment point near the muscle. The ground wire was placed between the stimulating electrode and the recording electrode. During motor conduction measurement, super-stimulation was applied to the nerve stem to increase the stimulation intensity by 10–30% to induce the maximum CMAP stimulation intensity. Stimulus duration was 0.1 or 0.2 ms.

The F-wave measurement method was similar to that of the motor conduction measurement, while the cathode of the stimulating electrode was placed at the proximal end.

The reference parameters of adult neurophysiology in the study laboratory were as follows. Median nerve: motor conduction: conduction velocity (MCV) of 50–60 m/s (wrist to elbow) and amplitude (CMAP) was ≥4–5 mV (wrist stimulation); sensory conduction: conduction velocity (SCV) of 50–65 m/s, amplitude (SNAP) of ≥20 μV, and F-wave latency of 25–32 ms (wrist stimulation). Ulnar nerve: motor conduction: MCV of 50–60 m/s (wrist to elbow) and CMAP of ≥4–5 mV (wrist stimulation); sensory conduction: SCV of 50–65 m/s and SNAP of ≥15 μV. Tibial nerve: motor conduction (ankle to popliteal fossa): MCV of 40–50 m/s, CMAP of ≥4 mV, and H-reflex latency of 28–35 ms (popliteal fossa stimulation). Common peroneal nerve: motor conduction (ankle to fibular head): MCV of 40–50 m/s and CMAP of ≥2 mV. Sural nerve: SCV of 40–50 m/s and SNAP of ≥10 μV. Skin sympathetic response (SSR): latency of 1.3–1.7 s (hand and foot recording).

Conduction velocity may decrease in the elderly (about 1 m/s/10 years), with the amplitude decreasing slightly with age. The occurrence of PNP, F-wave abnormality, and conduction velocity (CV) were measured by NCS. The changes in two measurements were also recorded for patients who completed both follow-ups. PNP was categorized into mononeuropathy and polyneuropathy (i.e., mono neuritis multiplex and polyneuropathy) according to the *European Standardized Telematic Tool to Evaluate Electrodiagnostic Methods* (18). The pathological type was categorized into demyelination, axon loss, and mixed types. Electrophysiological evidence of axonal loss included the reduced SNAP or CMAP with normal or slightly reduced CV and evidence of demyelination markedly slowed conduction velocity or prolonged distal latency (18). The categorization was made based on published guidelines (16, 17). F-wave abnormality was determined by prolonged F-wave latency or < 60% occurrence rate of F-wave. Dichotomous indexes were used to describe the overall occurrence of PNP, mononeuropathy, polyneuropathy, F-wave abnormality, and occurrence of different pathological classifications (demyelination, axonal loss, and a mixed type), different anatomical locations (proximal or distal), a different side of the body (left, right, and both), as well as the occurrence of each nerve injured.

After the NCS, participants were interviewed by trained medical staff and completed the consultation and a series of questionnaires. The Borg Rating Scale of Perceived Exertion was used to evaluate the degree of fatigue (18). The Patient Health Questionnaire was used to screen for depression, and the Generalized Anxiety Disorder scale was used to filter the anxiety symptoms. During the follow-up, symptoms after discharge, such as fever, sore throat, cough, and others, were recorded.

Qingjin Yiqi granules (QJYQ) is a recent traditional Chinese medicine (TCM) prescription for patients with COVID-19 and was used during the study period at the study hospital. It includes 16 herbs: Renshen (*Ginseng radix et rhizoma*), Maidong (*Ophiopogonis radix*), Wuweizi (*Schisandrae chinensis* fructus), Fuling (*Poria*), Banxia (*Pinelliae rhizoma*), Xuanshen (*Scrophulariae radix*), Cangzhu (*Atractylodis rhizoma*), Chenpi (*Citri reticulatae pericarpium*), Gancao (*Glycyrrhizae radix et rhizoma*), Chaihu (*Bupleuri radix*), Shengma (*Cimicifugae rhizoma*), Yiyiren (*Coicis semen*), Huangqin (*Scutellariae radix*), Mabiancao (*Verbenae herba*), Lugen (*Phragmitis rhizoma*), and Danzhuye (*Lophatheri herba*). These herbs are initially extracted with water, followed by concentration and spray-drying to powder, after which excipients are added with the final mixture pelletized using the dry granulation method. The patients received QJYQ orally at 10 g twice daily for 14 days.

The patients were grouped as PNP vs. non-PNP according to the occurrence of PNP, F-wave abnormality, or abnormal CV during follow-up. No matching was done between groups.

### Statistical analysis

Statistical analyses were performed using R (version 4.1.3) and SPSS (version 26.0, IBM Corp., Armonk, N.Y., United States). Categorical variables were presented as count (percentage) and continuous variables as mean ± standard deviation (SD) or median (interquartile range, IQR). The χ^2^ or Fisher’s exact test was used to compare the proportions; the t-test or Mann–Whitney U test was used for continuous variables where appropriate. Values between two visits were analyzed using the paired *t*-test or Wilcoxon signed-rank test was used when appropriate. Univariable and multivariable logistic regression analyses explored risk factors associated with PNP, mononeuropathy, polyneuropathy, and F-wave abnormality. Variables included in the analyses were mainly determined based on previous studies (19, 20). The selected variables were screened using univariable analyses. Those with *p*-values <0.05 were included in the multivariable model. In the multivariable analysis, the p-value threshold was adjusted as 0.05/4 = 0.0125 to account for type I error. Two-tailed *p* < 0.05 were considered statistically significant.

## Results

**Figure 1:**
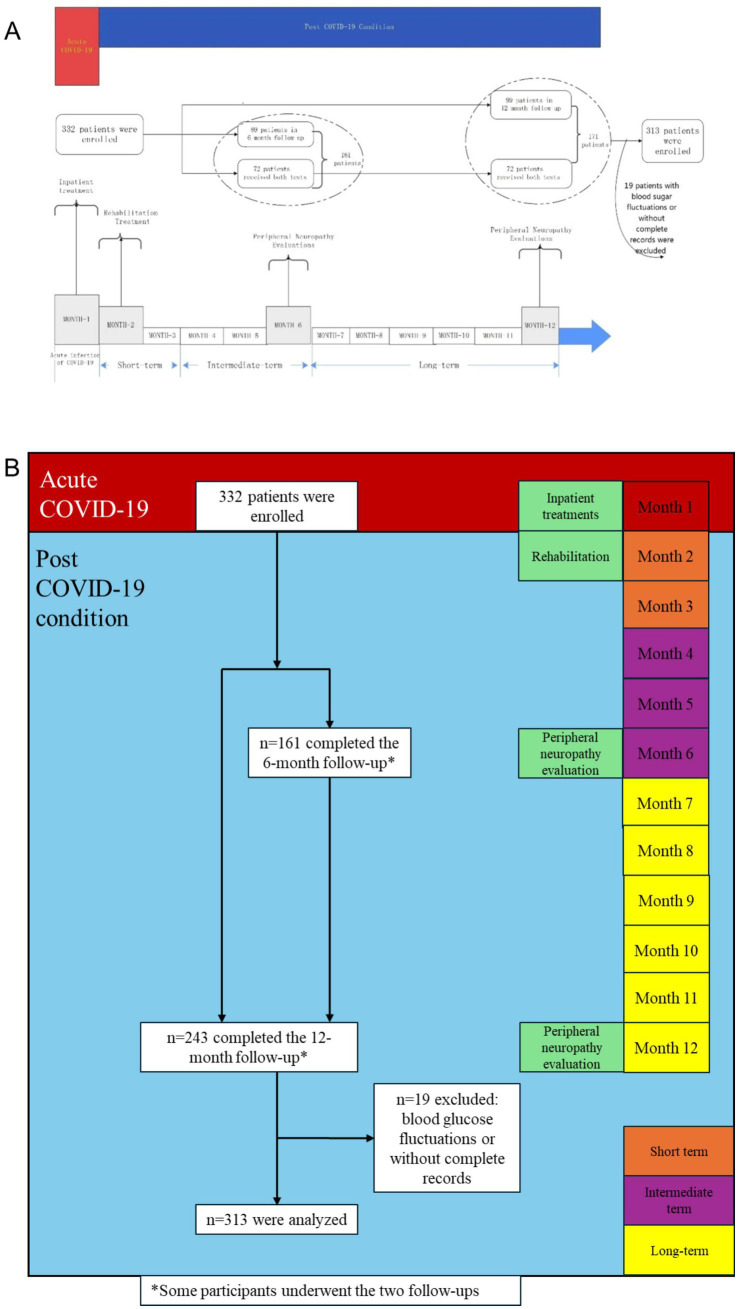
A framework and timeline of this study and patient flowchart. *Some participants underwent the two follow-ups.

A total of 332 participants were enrolled and received at least one NCS: 161 completed the 6-month follow-up, and 243 completed the 12-month follow-up. Nineteen patients with unstable blood glucose levels during follow-up or incomplete medical records or tests were excluded. Finally, 313 participants [aged 49.0 (IQR 33.0–58.0) years, and 191 (61.0%) females] were included for analysis, 150 of whom completed the 6-month follow-up assessment, 230 completed the 12-month follow-up assessment (Figure 1).

**Figure 2:**
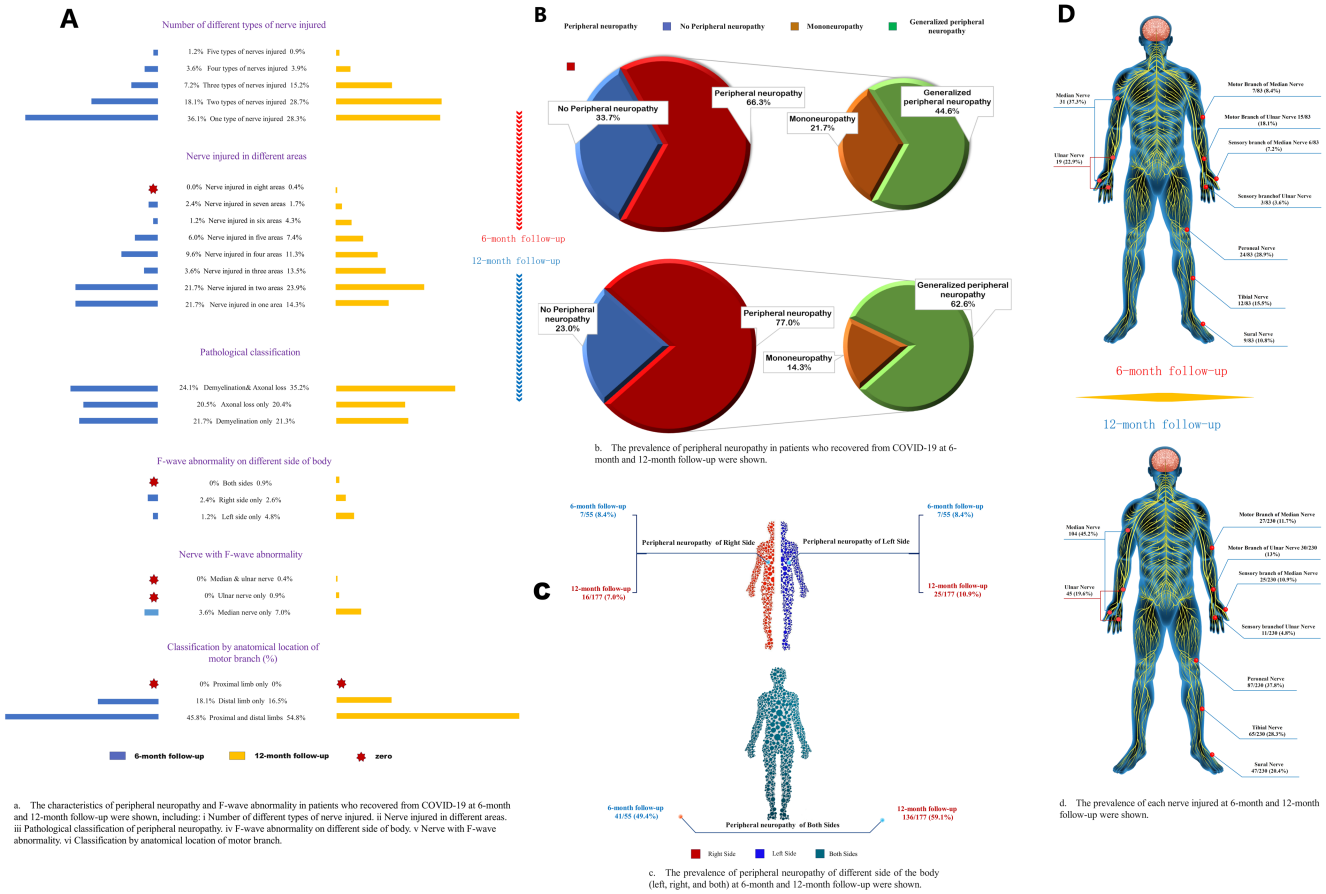
Characteristics of different subtypes of peripheral neuropathy. **(A)** The characteristics of peripheral neuropathy and F-wave abnormality in patients who recovered from COVID-19 at 6-month and 12-month follow-ups were shown, including (i) a number of different types of nerve injuries; (ii) nerve injuries in different areas; (iii) pathological classification of peripheral neuropathy; (iv) F-wave abnormality on different sides of body; (v) nerve with F-wave abnormality; (vi) classification by anatomical location of motor branch. **(B)** The frequency of peripheral neuropathy in patients who recovered from COVID-19 at 6-month and 12-month follow-up. **(C)** The frequency of peripheral neuropathy of different sides of the body (left, right, and both) at 6-month and 12-month follow-ups were shown. **(D)** The frequency of each nerve injured at 6-month and 12-month follow-ups were shown.

During the one-year follow-up period, 232 (74%) patients developed PNP (PNP group), while 81 (26%) did not (non-PNP group). The demographics and baseline characteristics between PNP and non-PNP groups were similar (all *p* < 0.05), except for age [43.0 (IQR 28.0–53.0) vs. 51.0 (IQR 34.8–59.0) years, *p* = 0.002] (Table 1). Multivariable logistic regression analysis showed that age [odds ratio (OR) = 1.22, confidence interval (CI): 1.05–1.41, *p* = 0.009] was independently associated with PNP (Table 2; Figure 2). Additionally, among patients in the PNP group, 51 (16%) patients had mononeuropathy, while 181 (58%) had polyneuropathy (Table 3).

**Table 1:** Characteristics of enrolled patients at baseline.

Variables	Total (*n* = 313)	No peripheral neuropathy (*n* = 81)	PNP (*n* = 232)	*p-*value
Age, mean ± SD, years	44.7 ± 17.5	39.8 ± 17.7	46.5 ± 17.1	0.003
median (IQR), years	49·0 (33.0–58.0)	43.0 (28.0–53.0)	51.0 (34.8–59.0)	0.002
Sex				0.12
Male	122 (39%)	38 (47%)	84 (36%)	
Female	191 (61%)	43 (53%)	148 (64%)	
Smoking				0.91
Never-smoker	283 (90%)	74 (91%)	209 (90%)	
Current or former smoker	30 (10%)	7 (9%)	23 (10%)	
Education				0.75
Middle school or lower	294 (94%)	75 (93%)	219 (94%)	
College or higher	19 (6%)	6 (7%)	13 (6%)	
Disease severity				0.25
Mild	96 (31%)	29 (36%)	67 (29%)	
Moderate	200 (64%)	50 (62%)	150 (65%)	
Severe or critically ill	17 (5%)	2 (3%)	15 (7%)	
Symptoms and signs at disease onset				
Fever	64 (20%)	16 (20%)	48 (21%)	0.98
Cough	70 (22%)	18 (22%)	52 (22%)	0.99
Dyspnea	16 (5%)	4 (5%)	12 (5%)	0.99
Fatigue	17 (5%)	2 (3%)	15 (7%)	0.28
Nausea or vomiting	9 (3%)	1 (1%)	8 (3%)	0.52
Diarrhea	5 (2%)	0 (0%)	5 (2%)	0.41
Myalgia	8 (3%)	1 (1%)	7 (3%)	0.64
Chest tightness	12 (4%)	2 (3%)	10 (4%)	0.68
Sputum production	33 (11%)	6 (7%)	27 (12%)	0.39
Comorbidity	78 (25%)	17 (21%)	61 (26%)	0.42
Hypertension	67 (21%)	15 (19%)	52 (22%)	0.56
Heart diseases	18 (6%)	5 (6%)	13 (6%)	0.99
Cerebrovascular diseases	4 (1%)	1 (1%)	3 (1%)	0.99
Chronic obstructive pulmonary diseases	5 (2%)	1 (1%)	4 (2%)	0.99
Antiviral therapy	32 (10%)	5 (6%)	27 (12%)	0.24
Corticosteroids therapy	7 (2%)	2 (3%)	5 (2%)	0.99
nCoV IgG at discharge, median (IQR), S/CO	31.8 (12.2–120.2)	23.1 (9.1–77.9)	35.6 (13.0–130.8)	0.08
Time from admission to follow-up, median (IQR), days	350.0 (165.0–357.0)	349.0 (154.0–356.0)	350.0 (324.5–357.0)	0.14
Length of hospital stay, median (IQR), days	17.0 (14.0–23.0)	17.0 (14.0–22.0)	17.0 (14.0–23.0)	0.48

The results are presented as means ± standard deviation, median [interquartile range (IQR)], or n (%), and, respectively, analyzed using Student’s t-test, Mann–Whitney U-test, and the chi-squared test.

**Table 2:** Univariable logistic regression analysis for peripheral neuropathy, mononeuropathy, polyneuropathy, and F-wave abnormality.

	Peripheral neuropathy		Mononeuropathy		Polyneuropathy		F-wave abnormality	
	OR (95%CI)	*p-*value	OR (95%CI)	*p-*value	OR (95%CI)	*p-*value	OR (95%CI)	*p-*value
Age^*^	1.24 (1.07–1.43)	0.004	1.00 (0.84–1.19)	>0.99	1.19 (1.04–1.35)	0.010	1.00 (0.78–1.29)	0.98
Sex								
Men	1 (ref)		1 (ref)		1 (ref)		1 (ref)	
Women	1.56 (0.93–2.60)	0.09	1.09 (0.59–2.06)	0.78	1.36 (0.86–2.15)	0.19	1.40 (0.57–3.76)	0.48
Education								
Middle school or lower	1 (ref)		1 (ref)		1 (ref)		1 (ref)	
College or higher	0.74 (0.28–2.18)	0.56	4.25 (1.56–11.10)	0.003	0.24 (0.08–0.64)	0.008	0.72 (0.04–3.78)	0.76
Smoking								
Never-smoker	1 (ref)		1 (ref)		1 (ref)		1 (ref)	
Current or former smoker	1.16 (0.50–3.03)	0.74	1.03 (0.33–2.63)	0.95	1.10 (0.52–2.44)	0.80	0.43 (0.02–2.18)	0.42
Comorbidity								
No	1 (ref)		1 (ref)		1 (ref)		1 (ref)	
Yes	1.34 (0.74–2.53)	0.34	0.80 (0.37–1.60)	0.55	1.42 (0.84–2.43)	0.20	1.14 (0.40–2.89)	0.79
nCoV IgG at discharge								
< median	1 (ref)		1 (ref)		1 (ref)		1 (ref)	
≥ median	1.46 (0.88–2.44)	0.15	1.38 (0.76–2.55)	0.30	1.12 (0.72–1.76)	0.61	0.35 (0.12–0.87)	0.03
Antiviral								
No	1 (ref)		1 (ref)		1 (ref)		1 (ref)	
Yes	2.00 (0.80–6.07)	0.17	1.51 (0.57–3.54)	0.37	1.24 (0.59–2.71)	0.57	0.40 (0.02–2.02)	0.38
Corticosteriods								
No	1 (ref)		1 (ref)		1 (ref)		1 (ref)	
Yes	0.87 (0.18–6.16)	0.87	0.85 (0.04–5.14)	0.88	0.97 (0.21–5.00)	0.97	2.26 (0.12–14.11)	0.46
Length of hospital stay								
≤14 days	1 (ref)		1 (ref)		1 (ref)		1 (ref)	
15–21 days	0.90 (0.49–1.65)	0.75	0.95 (0.45–2.03)	0.89	0.95 (0.55–1.63)	0.85	1.67 (0.59–5.40)	0.35
>21 days	1.16 (0.59–2.31)	0.67	1.23 (0.57–2.71)	0.60	0.99 (0.55–1.80)	0.98	0.98 (0.26–3.63)	0.97

*OR (95% CI) for age indicates the risk of outcome per 10-year age increase. OR, odds ratio. 95%CI, 95% confidence interval.

**Table 3:** Characteristics of peripheral neuropathy subtypes.

	Total (*n* = 313)	6-month follow-up (*n* = 83)	12-month follow-up (*n* = 230)	*p*-value
Peripheral neuropathy				0.080
Yes	232 (74%)	55 (66%)	177 (77%)	
No	81 (26%)	28 (34%)	53 (23%)	
Classification				
Mononeuropathy	51 (16%)	18 (22%)	33 (14%)	0.170
Polyneuropathy	181 (58%)	37 (45%)	144 (63%)	0.006
Nerve Injury				
Median nerve	135 (43%)	31 (37%)	104 (45%)	0.27
Different branch				0.55
Motor branch only	34 (11%)	7 (8%)	27 (12%)	
Sensory branch only	31 (10%)	6 (7%)	25 (11)	
Motor and Sensory	70 (22%)	18 (22%)	52 (23%)	
Ulnar nerve	64 (20%)	19 (23%)	45 (20%)	0.63
Different branch				0.69
Motor branch only	45 (14%)	15 (18%)	30 (13%)	
Sensory branch only	14 (5%)	3 (4%)	11 (5%)	
Motor and Sensory	5 (2%)	1 (1%)	4 (2%)	
Peroneal nerve	111 (36%)	24 (29%)	87 (38%)	0.19
Tibial nerve	77 (25%)	12 (15%)	65 (28%)	0.02
Sural nerve	56 (18%)	9 (11%)	47 (20%)	0.07
Nerve injuries in multiple areas				0.12
Nerve injured in one area	51 (16%)	18 (22%)	33 (14%)	
Nerve injury in two areas	73 (23%)	18 (22%)	55 (24%)	
Nerve injury in three areas	34 (11%)	3 (4%)	31 (14%)	
Nerve injury in four areas	34 (11%)	8 (10%)	26 (11%)	
Nerve injury in five areas	22 (7%)	5 (6%)	17 (7%)	
Nerve injury in six areas	11 (4%)	1 (1%)	10 (4%)	
Nerve injury in seven areas	6 (2%)	2 (2%)	4 (2%)	
Nerve injury in eight areas	1 (0%)	0 (0%)	1 (0%)	
Number of different types of nerve injuries*				0.09
One type of injury	95 (30%)	30 (36%)	65 (28%)	
Two types of injury	81 (26%)	15 (18%)	66 (29%)	
Three types of injury	41 (13%)	6 (7%)	35 (15%)	
Four types of injury	12 (4%)	3 (4%)	9 (4%)	
Five types of injury	3 (1%)	1 (1%)	2 (1%)	
Pathological classification				0.17
Demyelination only	67 (21%)	18 (22%)	49 (21%)	
Axonal loss only	64 (20%)	17 (21%)	47 (20%)	
Demyelination combined with axonal loss	101 (32%)	20 (24%)	81 (35%)	
Peripheral neuropathy on different sides of the body				0.24
Left side only	32 (10%)	7 (8%)	25 (11%)	
Right side only	23 (7%)	7 (8%)	16 (7%)	
Both sides	177 (57%)	41 (49%)	136 (59%)	
Classification by anatomical location of motor branch				
Proximal limb only	0 (0%)	0 (0%)	0 (0%)	-
Distal limb only	53 (17%)	15 (18%)	38 (17%)	0.88
Proximal and distal limbs	164 (52%)	38 (46%)	126 (55%)	0.20
F-wave abnormality	22 (7%)	3 (4%)	19 (8%)	0.24
F-wave abnormality on different sides of the body				0.41
Left side only	12 (4%)	1 (1%)	11 (5%)	
Right side only	8 (3%)	2 (2%)	6 (3%)	
Both sides	2 (1%)	0 (0%)	2 (1%)	
Nerve with F-wave abnormality				0.50
Median nerve only	19 (6%)	3 (4%)	16 (7%)	
Ulnar nerve only	2 (1%)	0 (0%)	2 (1%)	
Median and ulnar nerve	1 (0%)	0 (0%)	1 (0%)	

*One type of nerve contains the same nerve on both sides of body.

Next, multivariable logistic regression analysis showed that college or higher education (OR = 5.07, 95% CI: 1.80–13.90, *p* = 0.002) was independently associated with mononeuropathy, while no factors were associated with polyneuropathy.

**Figure 3:**
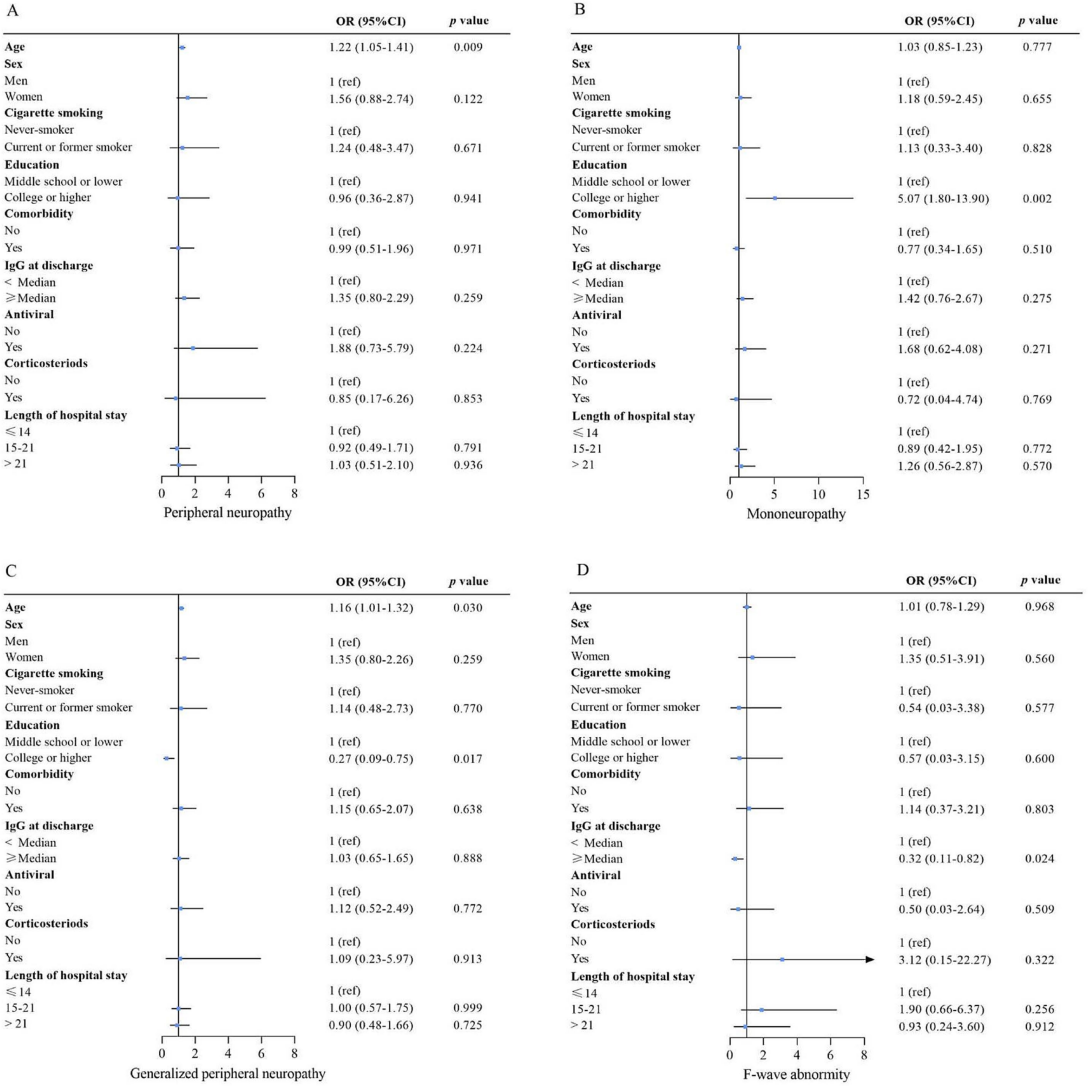
Risk factors associated with peripheral neuropathy **(A)**, mononeuropathy **(B)**, generalized peripheral neuropathy **(C)**, and F-wave abnormality **(D)**. For the association of nCoV IgG at discharge with outcome, age, sex, smoking, education, comorbidity, antiviral, corticosteroids, and length of hospital stay were adjusted. For the association of sex, antiviral, corticosteroid, and length of hospital stay with outcome, the variables above were all included in the model. Except for comorbidity, the variables mentioned above were included for the education association with the outcomes. The variables above, except for comorbidity, IgG at discharge, and length of hospital stay, were included for the association of smoking with the outcome. For the association of comorbidity with outcome, the variables above, except for IgG at discharge and length of hospital stay, were included for the association of age with outcome; only sex, cigarette smoking, and education were adjusted. OR (95% CI) for age indicates the outcome risk per 10-year age increase. Multivariable logistic regression analyses. OR = odds ratio. 95% CI = 95% confidence interval. The *p*-value threshold was adjusted as 0.05/4 = 0.0125 to account for type I error.

F-wave abnormalities were detected in 22 (7%) out of 313 patients (Table 3), but no factors were independently associated with F-wave abnormality (Table 2; Figure 3).

The occurrence of polyneuropathy [37 (45%) vs. 144 (63%), *p* = 0.006] and tibial nerve injury [12 (15%) vs. 65 (28%), *p* = 0.020] were higher at 12-month follow-up compared to 6-month follow-up (Figure 2; Supplementary Table S1). Eighty-one (26%), 41 (13%), 12 (4%), and 3 (1.0%) patients had two, three, four, and five types of nerve injury that co-occurred, respectively (Table 4). According to pathological classification, the peroneal nerve type of PNP at 12-month follow-up decreased compared to 6-month follow-up [5 (6%) vs. 3 (1%), *p* = 0.020; Table 5]. In addition, memory loss, found in 269 patients (85.9%; 74 patients with PNP and 195 without PNP), was the most commonly reported symptom (Table 6).

**Table 4:** Occurrence of peripheral neuropathy in two or more types of nerve injury.

	Total (*n* = 313)	6-month follow-up (*n* = 83)	12-month follow-up (*n* = 230)	*p-*value
Two nerve injuries that occurred simultaneously	81 (26%)	15 (18%)	66 (29%)	0.08
Median nerve & Ulnar nerve	10 (3%)	2 (2%)	8 (4%)	0.91
Median nerve & Peroneal nerve	21 (7%)	6 (7%)	15 (7%)	0.99
Median nerve & Tibial nerve	9 (3%)	1 (1%)	8 (4%)	0.50
Median nerve & Sural nerve	11 (4%)	1 (1%)	10 (4%)	0.32
Ulnar nerve & Peroneal nerve	5 (2%)	1 (1%)	4 (2%)	0.99
Ulnar nerve & Tibial nerve	4 (1%)	0 (0%)	4 (2%)	0.52
Ulnar nerve & Sural nerve	4 (1%)	1 (1%)	3 (1%)	0.99
Peroneal nerve & Tibial nerve	11 (4%)	2 (2%)	9 (4%)	0.77
Peroneal nerve & Sural nerve	5 (2%)	0 (0%)	5 (2%)	0.40
Tibial nerve & Sural nerve	1 (0%)	1 (1%)	0 (0%)	0.59
Three nerve injuries that occurred simultaneously	41 (13%)	6 (7%)	35 (15%)	0.10
Median nerve & Ulnar nerve & Peroneal nerve	5 (2%)	1 (1%)	4 (2%)	0.99
Median nerve & Ulnar nerve & Tibial nerve	3 (1%)	1 (1%)	2 (1%)	0.99
Median nerve & Ulnar nerve & Sural nerve	2 (1%)	1 (1%)	1 (0%)	0.99
Median nerve & Peroneal nerve & Tibial nerve	11 (4%)	0 (0%)	11 (5%)	0.09
Median nerve & Peroneal nerve & Sural nerve	8 (3%)	1 (1%)	7 (3%)	0.61
Median nerve &Tibial nerve& Sural nerve	4 (1%)	0 (0%)	4 (2%)	0.52
Ulnar nerve & Peroneal nerve & Tibial nerve	4 (1%)	1 (1%)	3 (1%)	0.99
Ulnar nerve & Peroneal nerve & Sural nerve	1 (0%)	1 (1%)	0 (0%)	0.59
Ulnar nerve & Tibial nerve & Sural nerve	1 (0%)	0 (0%)	1 (0%)	0.99
Peroneal nerve & Tibial nerve & Sural nerve	2 (1%)	0 (0%)	2 (1%)	0.96
Four nerve injuries that occurred simultaneously	12 (4%)	3 (4%)	9 (4%)	0.99
Five nerves, except for the median nerve	0 (0%)	0 (0%)	0 (0%)	-
Five nerves, except for the Tibial nerve	4 (1%)	1 (1%)	3 (1%)	0.99
Five nerves, except for the Peroneal nerve	1 (0%)	0 (0%)	1 (0%)	0.99
Five nerves, except for the Tibial nerve	3 (1%)	1 (1%)	2 (1%)	0.99
Five nerves, except for the Sural nerve	4 (1%)	1 (1%)	3 (1%)	0.99
Five nerve injuries that occurred simultaneously	3 (1%)	1 (1%)	2 (1%)	0.99

**Table 5:** Pathological classification of peripheral neuropathy of each nerve.

Pathological classification	Total (*n* = 313)	6-month follow-up (*n* = 83)	12-month follow-up (*n* = 230)	*p*-value
Median nerve				0.4
Demyelination only	130 (42%)	31 (37%)	99 (43%)	
Axonal loss only	2 (1%)	0 (0%)	2 (1%)	
Demyelination combined with axonal loss	3 (1%)	0 (0%)	3 (1%)	
Ulnar nerve				0.44
Demyelination only	14 (5%)	3 (4%)	11 (5%)	
Axonal loss only	47 (15%)	16 (19%)	31 (14%)	
Demyelination combined with axonal loss	3 (1%)	0 (0%)	3 (1%)	
Peroneal nerve				0.02
Demyelination only	8 (3%)	5 (6%)	3 (1%)	
Axonal loss only	96 (31%)	17 (21%)	79 (34%)	
Demyelination combined with axonal loss	7 (2%)	2 (2%)	5 (2%)	
Tibial nerve				0.06
Demyelination only	16 (5%)	2 (2%)	14 (6%)	
Axonal loss only	55 (18%)	8 (10%)	47 (20%)	
Demyelination combined with axonal loss	6 (2%)	2 (2%)	4 (2%)	
Sural nerve^*^				0.16
Demyelination only	53 (17%)	8 (10%)	45 (20%)	
Axonal loss only	1 (0%)	0 (0%)	1 (0%)	
Demyelination combined with axonal loss	1 (0%)	0 (0%)	1 (0%)	

*Both sides of the sural nerves were not detected in one patient.

**Table 6:** Symptoms after discharge in COVID-19 patients.

Symptoms	Total (*n* = 313)	No peripheral neuropathy (*n* = 81)	Peripheral neuropathy (*n* = 232)	*p-*value
Fever	2 (1%)	1 (1%)	1 (0%)	0.99
Cough	55 (18%)	16 (20%)	39 (17%)	0.67
Expectoration	45 (14%)	12 (15%)	33 (14%)	0.99
Dry throat	65 (21%)	20 (25%)	45 (19%)	0.39
Pharyngeal itching	36 (12%)	11 (14%)	25 (11%)	0.63
Congestion in the throat	33 (11%)	9 (11%)	24 (10%)	0.99
Sore throat	14 (5%)	6 (7%)	8 (3%)	0.24
Heterorexia	9 (3%)	2 (3%)	7 (3%)	0.99
Olfactory abnormality	24 (8%)	9 (11%)	15 (7%)	0.27
Muscle weakness	66 (21%)	14 (17%)	52 (22%)	0.41
Hyperhidrosis	51 (16%)	14 (17%)	37 (16%)	0.92
Aversion to wind	52 (17%)	13 (16%)	39 (17%)	0.99
Shortness of breath	56 (18%)	14 (17%)	42 (18%)	0.99
Chest tightness	47 (15%)	11 (14%)	36 (16%)	0.81
Chest pain	12 (4%)	3 (4%)	9 (4%)	0.99
Physical pain	37 (12%)	11 (14%)	26 (11%)	0.71
Palpitation	45 (14%)	10 (12%)	35 (15%)	0.67
Dysphoria	75 (24%)	19 (24%)	56 (24%)	0.99
Sleep disorders	74 (24%)	18 (22%)	56 (24%)	0.84
Hair loss	86 (28%)	23 (28%)	63 (27%)	0.94
Dizziness	41 (13%)	10 (12%)	31 (13%)	0.97
Headache	29 (9%)	10 (12%)	19 (8%)	0.37
Weakness of lower limbs	37 (12%)	8 (10%)	29 (13%)	0.67
Bitter taste	65 (21%)	14 (17%)	51 (22%)	0.46
Nausea	12 (4%)	4 (5%)	8 (3%)	0.79
Decreased appetite	12 (4%)	2 (3%)	10 (4%)	0.68
Constipation	38 (12%)	5 (6%)	33 (14%)	0.09
Diarrhea	21 (7%)	7 (9%)	14 (6%)	0.58
Abnormal urination	23 (7%)	4 (5%)	19 (8%)	0.47
Fatigue	63 (20%)	13 (16%)	50 (22%)	0.37
Memory Loss	269 (86%)	74 (91%)	195 (84%)	0.15

## Discussion

In this study, we assessed the risk factors for PNP and F-wave abnormality during 1 year of COVID-19 rehabilitation in 313 participants, 232 (74%) of whom developed PNP. Our data suggested that age might be an independent risk factor for PNP, while higher education appears to be a risk factor for mononeuropathy. Also, the level of nCoV IgG at discharge might be an independent risk factor for F-wave abnormality during one-year rehabilitation from COVID-19. These findings might help clinicians formulate clinical rehabilitation directions for COVID-19 survivors.

The present study suggests that those with high education levels are at an increased risk of mononeuropathy. The literature suggests no direct, strong association between education level and the development of mononeuropathy (or polyneuropathy), but a lower education level may be associated with a higher prevalence of painful diabetic peripheral neuropathy (DPN) (21–24). Additional studies will be necessary to examine the issue. Nevertheless, we found that demyelination of the median nerve motor branch and the sensory branch is a common injury, suggesting that these patients are likely to have carpal tunnel syndrome (CTS). Causes of CTS include obesity, contraction of the wrist or repetitive flexion, wrist-related occupations, diabetes, and potential PNP leading to hypertrophy (25). We also compared the characteristics of PNP at two visits, finding that demyelination of the sensory and motor branches of the median nerve was aggravated, suggesting that COVID-19 may aggravate and lead to CTS.

The involvement of the central and peripheral nervous systems can have several pathogenic pathways, including endothelial dysfunction, direct viral lesions, thrombotic microangiopathy, hypoxia, systemic inflammation, and autoimmune reactions. In SARS-CoV-2 infection, neuroinflammatory changes of the brain and brainstem are the most common autopsy findings. Acute neurologic manifestations are more often present in the severe form of COVID-19 and are linked to poor prognosis (26).

Previous studies (20, 27, 28) assessed 1-year outcomes in hospital survivors with COVID-19, finding that most patients had at least one sequelae symptom 6 months after discharge, which is consistent with our data. However, no significant association between PNP and symptoms was observed in this study, highlighting the need to focus on peripheral nerves during follow-up of all COVID-19 survivors. Several non-respiratory manifestations were reported after COVID-19 (29, 30), and the occurrence of PNP accounts for 7.2–42.8% (31, 32). In the present study, the frequency of PNP was 74%, higher than the reported range. The higher frequency could be related to a selection bias since only patients hospitalized for COVID-19 were included, possibly shifting the disease severity distribution toward more severe disease. Different criteria for PNP among studies could also account for the differences in frequency.

Nevertheless, the specific mechanism of PNP after COVID-19 remains unclear. The current research focuses on three aspects. (1) Autoimmune response. Finsterer et al. (9) suggested that SARS-CoV-2 may have epitopes similar to peripheral nerve components (activating autoreactive B or T cells), resulting in PNP. SARS-CoV-2 spikes interact with GM1 gangliosides in peripheral nerves, which leads to cross-reactivity and the production of antibodies against antigens, inducing a peripheral demyelinating pattern of GBS (33). (2) Direct neuroinvasive effects of SARS-CoV-2. The nervous system damage of COVID-19 patients is commonly caused by acute nervous system infection (7, 34). Infection with SARS-CoV-2 increases the risk of neurological damage. In addition, SARS-CoV-2 can lead to exacerbate pre-existing baseline neurological disease severity or acute neurological damage in some patients. Another factor to consider is the possible direct neuro-muscular damage caused by the SARS-CoV-2 virus. However, direct muscle damage by the virus has not been demonstrated in autologous muscle lesions. (3) Persistent and recurring neuroinflammatory responses and damage. Immune dysregulation after infection with SARS-CoV-2 may persist in the form of persistent inflammation, immunosuppression, catabolic syndrome, and immunosuppression (35). The cytokine storm could also be involved (36). This immune state is triggered by cytokine storms during acute infection and is further promoted by the sustained release of endogenous alarmins or danger-associated molecular patterns, which can lead to chronic systemic inflammation. Chronic neuroinflammation associated with high levels of cytokines/chemokines may be involved in the pathogenesis and progression of neurological diseases (35).

In this study, the mixed type (axonal loss combined with demyelination) had the highest rate among the pathological types. The median nerve injury was the most common. In addition, sensory and motor branches were often damaged, with demyelination and distal (carpal-palm) involvement, followed by the peroneal nerve with mainly axonal loss in both proximal and distal ends. Compared to 6-month follow-up data, the sural and ulnar nerve amplitude increased at the 12-month follow-up, while the median nerve conduction velocity decreased in patients who completed two measurements. These results suggest that the demyelination of the median nerve was aggravated, and axonal loss of the sural and the ulnar nerves was relieved. Studies have found that COVID-19 patients are more at risk of having PNP in the distal limb and higher occurrence in the lower extremities, especially when undergoing long-term rehabilitation (37, 38). Thus, for COVID-19 patients, especially older adults with low IgG antibody levels, more attention should be given to the peripheral nerve condition. Early NCS screening and prophylactic administration of vitamin B6 and mecobalamin may be recommended for these patients (39, 40).

Electrodiagnostic studies are very beneficial when evaluating patients with suspected PNP (41, 42). In this study, NCS was performed using full-featured electromyography/evoked potential equipment during the follow-up. Most patients had PNP, which was consistent with previous studies. Oaklander et al. (43) evaluated the PNP of 17 patients with prolonged long COVID-19 and found that 59% of cases had ≥1 test interpretation confirming neuropathy. Moreover, another study (44) reviewed 143 studies reporting on nervous system involvement by COVID-19, detecting central and peripheral nervous system involvement in 10,723 COVID-19 patients. The study reflected the remarkable occurrence of neurological involvement in COVID-19 patients, ranging from 22.5 to 36.4%. However, previous studies on the nervous system have mainly focused on the central nervous system (45–47); also, most were case reports or focused mainly on severe COVID-19 patients (48). Finsterer et al. (9) analyzed 105 retrieved articles on SARS-CoV-2-related PNP, including 220 patients with and 41 patients without Guillain-Barre syndrome (GBS) neuropathy. A total of 168 patients reported latency between viral infection and the onset of neuropathy, ranging from −10 to 90 days. In addition, neuropathy was classified in 257 patients. Moreover, 220 patients were diagnosed with GBS, 11 with mononeuritis multiple neuropathy, 4 with severe neuropathy, 16 with plexus neuropathy, 4 with isolated sensory neuropathy, and 2 with leprosy. A review highlighted the association between GBS and COVID-19, suggesting that GBS could be caused by COVID-19 in these patients (49). COVID-19 appears to cause the senescence of dopaminergic neurons (50). However, electrophysiological testing was not performed in the studies mentioned above. PNP is relatively insidious, and the clinical manifestations are not typical (51). Many cases tend to develop PNP after acute infection (37). Also, there is a lack of large-scale studies assessing PNP in COVID-19 patients, especially those investigating neurological recovery in long-term rehabilitation of COVID-19 survivors.

NCS is important for diagnosing PNP and selecting the best therapy (52). A previous study (53) assessed the correlation between electromyography and NCS in COVID-19 patients and found that NCS is important for predicting COVID-19 prognosis and recovery. Using NCS, we found that most patients had persistent PNP, mainly polyneuropathy, 6 or 12 months after infection with SARS-CoV-2. Also, PNP was positively associated with age. The occurrence of F-wave abnormality was relatively low.

The present study has several limitations. First, some symptoms, such as fatigue, nausea, and chest tightness, were self-reported, which may lead to recall bias. Second, not all enrolled patients were measured twice, and the NCS results of patients at 6-month follow-up may change after that, which may give rise to an attrition bias. The positive patients were hospitalized at the same place, and activities were severely limited during the lockdown, but after 6–12 months and once the lockdown was lifted, the recovered patients resumed school and work or returned to their living place. Still, those patients were included in the study on the basis that they would consult if neurological symptoms occurred. A selection bias is also unavoidable since selection criteria were applied when enrolling the patients. Third, the relatively small proportion of severe or critically severe COVID-19 patients in our study limits the generalizability of the findings. The study population was limited to a single hospital in China, which may not be generalizable to populations with different demographics, healthcare access, and genetic predispositions. Fourth, this study did not describe the intrinsic link between PNP and persistent multi-organ functional injury. nCoV IgG levels were measured at discharge, while the development of F-wave abnormalities was assessed at 6 and 12 months. The IgG levels were not measured during follow-up, and no dynamic data were available after discharge. In addition, the exact moment when F-wave abnormalities developed is also unknown. Fifth, some risk factors for PNP were not accounted for, such as diabetes, lifestyle factors, and vitamin deficiencies. Sixth, the present study used the cut-offs suggested by Tankisi et al. in 2005 (18), but the selection of the cut-off can affect the results and conclusions. Finally, the use of ORs can overestimate the risk, especially when the frequency of the outcome is high. Next, we plan to analyze more clinical data of COVID-19 patients during hospitalization and discharge; blood biochemical test indicators, six-minute walk tests, lung function, anxiety/depression, etc., will be assessed during this phase. Correlation analysis with the relevant indicators of peripheral nerve electrophysiology will be carried out to fully elucidate the relationship between PNP and the function of major organs of the body and further study the pathophysiological mechanism of PNP to provide objective data for complete recovery. Nevertheless, these results suggest that older patients with COVID-19 and those with high nCoV IgG levels at discharge should be monitored for PNP during follow-up. Future studies should be performed with larger numbers of patients to confirm those factors and determine their exact predictive value for PNP and other abnormalities.

In conclusion, age might be an independent risk factor for PNP, while higher education was associated with mononeuropathy. Future studies should investigate the factors and interventions for PNP in patients with COVID-19.

## Data Availability

The original contributions presented in the study are included in the article/Supplementary material, further inquiries can be directed to the corresponding authors.

## References

[1] Varatharaj A Thomas N Ellul MA Davies NWS Pollak TA Tenorio EL. Neurological and neuropsychiatric complications of Covid-19 in 153 patients: a Uk-wide surveillance study. Lancet Psychiatry. (2020) 7:875–82. doi: 10.1016/s2215-0366(20)30287-x, PMID: 32593341 PMC7316461

[2] Zubair AS McAlpine LS Gardin T Farhadian S Kuruvilla DE Spudich S. Neuropathogenesis and neurologic manifestations of the coronaviruses in the age of coronavirus disease 2019: a review. JAMA Neurol. (2020) 77:1018–27. doi: 10.1001/jamaneurol.2020.2065, PMID: 32469387 PMC7484225

[3] Saif DS Ibrahem RA Eltabl MA. Prevalence of peripheral neuropathy and myopathy in patients post-Covid-19 infection. Int J Rheum Dis. (2022) 25:1246–53. doi: 10.1111/1756-185x.14409, PMID: 35915515 PMC9538868

[4] Pons-Escoda A Naval-Baudín P Majós C Camins A Cardona P Cos M. Neurologic involvement in Covid-19: cause or coincidence? A neuroimaging perspective. AJNR Am J Neuroradiol. (2020) 41:1365–9. doi: 10.3174/ajnr.A6627, PMID: 32527842 PMC7658883

[5] Mao L Jin H Wang M Hu Y Chen S He Q. Neurologic manifestations of hospitalized patients with coronavirus disease 2019 in Wuhan, China. JAMA Neurol. (2020) 77:683–90. doi: 10.1001/jamaneurol.2020.1127, PMID: 32275288 PMC7149362

[6] Watson JC Dyck PJ. Peripheral neuropathy: a practical approach to diagnosis and symptom management. Mayo Clin Proc. (2015) 90:940–51. doi: 10.1016/j.mayocp.2015.05.004, PMID: 26141332

[7] Jha NK Ojha S Jha SK Dureja H Singh SK Shukla SD. Evidence of coronavirus (Cov) pathogenesis and emerging pathogen Sars-Cov-2 in the nervous system: a review on neurological impairments and manifestations. J Molecular Neurosci: MN. (2021) 71:2192–209. doi: 10.1007/s12031-020-01767-6, PMID: 33464535 PMC7814864

[8] Wang S Liu X Wang Y. Evaluation of platelet-rich plasma therapy for peripheral nerve regeneration: a critical review of literature. Front Bioeng Biotechnol. (2022) 10:808248. doi: 10.3389/fbioe.2022.808248, PMID: 35299637 PMC8923347

[9] Finsterer J Scorza FA Scorza CA Fiorini AC. Peripheral neuropathy in Covid-19 is due to immune-mechanisms, pre-existing risk factors, anti-viral drugs, or bedding in the intensive care unit. Arq Neuropsiquiatr. (2021) 79:924–8. doi: 10.1590/0004-282x-anp-2021-0030, PMID: 34287509

[10] Aiyegbusi OL Hughes SE Turner G Rivera SC McMullan C Chandan JS. Symptoms, complications and Management of Long Covid: a review. J R Soc Med. (2021) 114:428–42. doi: 10.1177/01410768211032850, PMID: 34265229 PMC8450986

[11] Gupta A Madhavan MV Sehgal K Nair N Mahajan S Sehrawat TS. Extrapulmonary manifestations of Covid-19. Nat Med. (2020) 26:1017–32. doi: 10.1038/s41591-020-0968-3, PMID: 32651579 PMC11972613

[12] Qiu Y Li H Yang Z Liu Q Wang K Li R. The prevalence and economic burden of pain on middle-aged and elderly Chinese people: results from the China health and retirement longitudinal study. BMC Health Serv Res. (2020) 20:600. doi: 10.1186/s12913-020-05461-6, PMID: 32611450 PMC7329515

[13] Scheidl E Canseco DD Hadji-Naumov A Bereznai B. Guillain-barré syndrome during Sars-cov-2 pandemic: a case report and review of recent literature. J Peripher Nerv Syst. (2020) 25:204–7. doi: 10.1111/jns.12382, PMID: 32388880 PMC7273104

[14] Abolmaali M Heidari M Zeinali M Moghaddam P Ramezani Ghamsari M Jamshidi Makiani M. Guillain-Barré syndrome as a Parainfectious manifestation of Sars-Cov-2 infection: a case series. J Clin Neurosci: Official J Neurosurg Society of Australasia. (2021) 83:119–22. doi: 10.1016/j.jocn.2020.11.013, PMID: 33281050 PMC7666532

[15] Yang Q Durmer JL Wheaton AG Jackson SL Zhang Z. Sleep duration and excess heart age among us adults. Sleep Health. (2018) 4:448–55. doi: 10.1016/j.sleh.2018.07.001, PMID: 30241660 PMC10913064

[16] Stålberg E van Dijk H Falck B Kimura J Neuwirth C Pitt M. Standards for quantification of Emg and Neurography. Clin Neurophysiol: Official J Int Federation Clin Neurophysiol. (2019) 130:1688–729. doi: 10.1016/j.clinph.2019.05.008, PMID: 31213353

[17] Tankisi A Pedersen TH Bostock H Z'Graggen WJ Larsen LH Meldgaard M. Early detection of evolving critical illness myopathy with muscle velocity recovery cycles. Clin Neurophysiol. (2021) 132:1347–57. doi: 10.1016/j.clinph.2021.01.017, PMID: 33676846

[18] Tankisi H Pugdahl K Fuglsang-Frederiksen A Johnsen B de Carvalho M Fawcett PR. Pathophysiology inferred from Electrodiagnostic nerve tests and classification of polyneuropathies. Suggested guidelines. Clin Neurophysiol: Official J Int Federation Clin Neurophysiol. (2005) 116:1571–80. doi: 10.1016/j.clinph.2005.04.003, PMID: 15907395

[19] Huang C Huang L Wang Y Li X Ren L Gu X. 6-month consequences of Covid-19 in patients discharged from hospital: a cohort study. Lancet (London, England). (2021) 397:220–32. doi: 10.1016/s0140-6736(20)32656-8, PMID: 33428867 PMC7833295

[20] Huang L Yao Q Gu X Wang Q Ren L Wang Y. 1-year outcomes in hospital survivors with Covid-19: a longitudinal cohort study. Lancet (London, England). (2021) 398:747–58. doi: 10.1016/s0140-6736(21)01755-4, PMID: 34454673 PMC8389999

[21] Bruce SG Young TK. Prevalence and risk factors for neuropathy in a Canadian first nation community. Diabetes Care. (2008) 31:1837–41. doi: 10.2337/dc08-0278, PMID: 18509208 PMC2518355

[22] Tumusiime DK Venter F Musenge E Stewart A. Prevalence of peripheral neuropathy and its associated demographic and health status characteristics, among people on antiretroviral therapy in Rwanda. BMC Public Health. (2014) 14:1306. doi: 10.1186/1471-2458-14-1306, PMID: 25526665 PMC4320525

[23] Li C Wang W Ji Q Ran X Kuang H Yu X. Prevalence of painful diabetic peripheral neuropathy in type 2 diabetes mellitus and diabetic peripheral neuropathy: a Nationwide cross-sectional study in mainland China. Diabetes Res Clin Pract. (2023) 198:110602. doi: 10.1016/j.diabres.2023.110602, PMID: 36871876

[24] Borbjerg MK Wegeberg AM Nikontovic A Mørch CD Arendt-Nielsen L Ejskjaer N. Understanding the impact of diabetic peripheral neuropathy and neuropathic pain on quality of life and mental health in 6,960 people with diabetes. Diabetes Care. (2025) 48:588–95. doi: 10.2337/dc24-2287, PMID: 39932781

[25] Chung T Prasad K Lloyd TE. Peripheral neuropathy: clinical and electrophysiological considerations. Neuroimaging Clin N Am. (2014) 24:49–65. doi: 10.1016/j.nic.2013.03.023, PMID: 24210312 PMC4329247

[26] Cavallieri F Sellner J Zedde M Moro E. Neurologic complications of coronavirus and other respiratory viral infections. Handb Clin Neurol. (2022) 189:331–58. doi: 10.1016/b978-0-323-91532-8.00004-5, PMID: 36031313 PMC9418023

[27] Davis HE McCorkell L Vogel JM Topol EJ. Long Covid: major findings, mechanisms and recommendations. Nat Rev Microbiol. (2023) 21:133–46. doi: 10.1038/s41579-022-00846-2, PMID: 36639608 PMC9839201

[28] Griffin DO. Postacute sequelae of Covid (Pasc or long Covid): an evidenced-based approach. Open Forum Infect Dis. (2024) 11:727. doi: 10.1093/ofid/ofae462, PMID: 39220656 PMC11363684

[29] Tsai PH Lai WY Lin YY Luo YH Lin YT Chen HK. Clinical manifestation and disease progression in Covid-19 infection. J Chin Med Assoc. (2021) 84:3–8. doi: 10.1097/jcma.0000000000000463, PMID: 33230062 PMC12965989

[30] Ricchio M Tassone B Pelle MC Mazzitelli M Serapide F Fusco P. Characteristics, management, and outcomes of elderly patients with diabetes in a Covid-19 unit: lessons learned from a pilot study. Medicina (Kaunas). (2021) 57:734. doi: 10.3390/medicina57040341, PMID: 33916210 PMC8065491

[31] Shahrizaila N Lehmann HC Kuwabara S. Guillain-barré syndrome. Lancet. (2021) 397:1214–28. doi: 10.1016/s0140-6736(21)00517-1, PMID: 33647239

[32] Malengreaux C Minguet P Colson C Dardenne N Misset B Rousseau AF. Incidence and risk factors of peripheral nerve injuries 3 months after ICU discharge: a retrospective study comparing Covid-19 and non-Covid-19 critically ill survivors. J Anesth Analg Crit Care. (2024) 4:10. doi: 10.1186/s44158-024-00144-8, PMID: 38336831 PMC10858596

[33] Dalakas MC. Guillain-Barré syndrome: the first documented Covid-19-triggered autoimmune neurologic disease: more to come with myositis in the offing. Neurology(R) Neuroimmunol Neuroinflammation. (2020) 7:783. doi: 10.1212/nxi.0000000000000781, PMID: 32518172 PMC7309518

[34] Saxena A Mautner J. A disease hidden in plain sight: pathways and mechanisms of neurological complications of post-acute sequelae of Covid-19 (Nc-Pasc). Mol Neurobiol. (2025) 62:2530–47. doi: 10.1007/s12035-024-04421-z, PMID: 39133434

[35] Groff D Sun A Ssentongo AE Ba DM Parsons N Poudel GR. Short-term and long-term rates of Postacute sequelae of Sars-Cov-2 infection: a systematic review. JAMA Netw Open. (2021) 4:e2128568. doi: 10.1001/jamanetworkopen.2021.28568, PMID: 34643720 PMC8515212

[36] Andersson U Tracey KJ. Vagus nerve Sars-Cov-2 infection and inflammatory reflex dysfunction: is there a causal relationship? J Intern Med. (2024) 295:91–102. doi: 10.1111/joim.13746, PMID: 38018736

[37] Balbi P Saltalamacchia A Lullo F Fuschillo S Ambrosino P Moretta P. Peripheral neuropathy in patients recovering from severe Covid-19: a case series. Medicina (Kaunas). (2022) 58:1090. doi: 10.3390/medicina58040523, PMID: 35454362 PMC9032555

[38] Ahmed ZB Razu MH Akter F Rabby MRI Karmaker P Khan M. Seropositivity of Sars-Cov-2 igg antibody among people in Dhaka City during the Prevaccination period. Biomed Res Int. (2022) 2022:4451144. doi: 10.1155/2022/4451144, PMID: 35097117 PMC8793344

[39] Brisset M Nicolas G. Peripheral neuropathies and aging. Geriatrie et Psychologie Neuropsychiatrie du Vieillissement. (2018) 16:409–13. doi: 10.1684/pnv.2018.0768, PMID: 30563801

[40] Ang CD Alviar MJ Dans AL Bautista-Velez GG Villaruz-Sulit MV Tan JJ. Vitamin B for treating peripheral neuropathy. Cochrane Database Syst Rev. (2008) 3:Cd004573. doi: 10.1002/14651858.CD004573.pub3 PMC12373429 18646107

[41] Foresti C Servalli MC Frigeni B Rifino N Storti B Gritti P. Covid-19 provoking Guillain-Barré syndrome: the Bergamo case series. Eur J Neurol. (2021) 28:e84–5. doi: 10.1111/ene.14549, PMID: 32961593 PMC7537113

[42] Abu-Rumeileh S Abdelhak A Foschi M Tumani H Otto M. Guillain-Barré syndrome Spectrum associated with Covid-19: an up-to-date systematic review of 73 cases. J Neurol. (2021) 268:1133–70. doi: 10.1007/s00415-020-10124-x, PMID: 32840686 PMC7445716

[43] Oaklander AL Mills AJ Kelley M Toran LS Smith B Dalakas MC. Peripheral neuropathy evaluations of patients with prolonged long Covid. Neurology(R) Neuroimmunol Neuroinflammation. (2022) 9:1140. doi: 10.1212/nxi.0000000000001146, PMID: 35232750 PMC8889896

[44] Guerrero JI Barragán LA Martínez JD Montoya JP Peña A Sobrino FE. Central and peripheral nervous system involvement by Covid-19: a systematic review of the pathophysiology, clinical manifestations, neuropathology, neuroimaging, electrophysiology, and cerebrospinal fluid findings. BMC Infect Dis. (2021) 21:515. doi: 10.1186/s12879-021-06185-6, PMID: 34078305 PMC8170436

[45] Dehghani A Zokaei E Kahani SM Alavinejad E Dehghani M Meftahi GH. The potential impact of Covid-19 on Cns and psychiatric sequels. Asian J Psychiatr. (2022) 72:103097. doi: 10.1016/j.ajp.2022.103097, PMID: 35405524 PMC8982477

[46] Beach SR Praschan NC Hogan C Dotson S Merideth F Kontos N. Delirium in Covid-19: a case series and exploration of potential mechanisms for central nervous system involvement. Gen Hosp Psychiatry. (2020) 65:47–53. doi: 10.1016/j.genhosppsych.2020.05.008, PMID: 32470824 PMC7242189

[47] Baig AM Khaleeq A Ali U Syeda H. Evidence of the Covid-19 virus targeting the Cns: tissue distribution, host-virus interaction, and proposed neurotropic mechanisms. ACS Chem Neurosci. (2020) 11:995–8. doi: 10.1021/acschemneuro.0c00122, PMID: 32167747

[48] Zhao H Shen D Zhou H Liu J Chen S. Guillain-Barré syndrome associated with Sars-Cov-2 infection: causality or coincidence? Lancet Neurol. (2020) 19:383–4. doi: 10.1016/s1474-4422(20)30109-5, PMID: 32246917 PMC7176927

[49] Pimentel V Luchsinger VW Carvalho GL Alcará AM Esper NB Marinowic D. Guillain-Barré syndrome associated with Covid-19: a systematic review. Brain Behav Immun-Health. (2023) 28:100578. doi: 10.1016/j.bbih.2022.100578 36686624 PMC9842533

[50] Yang L Kim TW Han Y Nair MS Harschnitz O Zhu J. Sars-Cov-2 infection causes dopaminergic neuron senescence. Cell Stem Cell. (2024) 31:196–211.e6. doi: 10.1016/j.stem.2023.12.012, PMID: 38237586 PMC10843182

[51] Marchettini P Lacerenza M Mauri E Marangoni C. Painful peripheral neuropathies. Curr Neuropharmacol. (2006) 4:175–81. doi: 10.2174/157015906778019536, PMID: 18615140 PMC2430688

[52] Tankisi H Pugdahl K Beniczky S Andersen H Fuglsang-Frederiksen A. Evidence-based recommendations for examination and diagnostic strategies of polyneuropathy Electrodiagnosis. Clin Neurophysiol Pract. (2019) 4:214–22. doi: 10.1016/j.cnp.2019.10.005, PMID: 31886447 PMC6921232

[53] Arkhipova-Jenkins I Helfand M Armstrong C Gean E Anderson J Paynter RA. Antibody response after Sars-Cov-2 infection and implications for immunity: a rapid living review. Ann Intern Med. (2021) 174:811–21. doi: 10.7326/m20-7547, PMID: 33721517 PMC8025942

[54] Zhao Y Yang Q Sun F Zhang M Lai Y Liu X. Covid-19 could cause long term peripheral nerve demyelination and axonal loss: a one year prospective cohort study. (2022).

