# Supplementary material for: Risk factors for 1-year peripheral neuropathy and F-wave abnormalities after COVID-19: a prospective cohort study
Source: Front Neurol. 2025 Jul 8;16:1532046. doi: 10.3389/fneur.2025.1532046 (PMC12282248; doi:10.3389/fneur.2025.1532046)
Supplement: Supplementary file 1 [file Table_1.docx]

**Table S1.** Characteristics of peripheral neuropathy subtypes

|  | **Total**  **（n=313）** | **6-month follow-up (n=83)** | **12-month follow-up (n=230)** | **p-value** |
| --- | --- | --- | --- | --- |
| **Peripheral neuropathy** |  |  |  | 0.080 |
| Yes | 232 (74%) | 55 (66%) | 177 (77%) |  |
| No | 81 (26%) | 28 (34%) | 53 (23%) |  |
| **Classification** |  |  |  |  |
| Mononeuropathy | 51 (16%) | 18 (22%) | 33 (14%) | 0.170 |
| Polyneuropathy | 181 (58%) | 37 (45%) | 144 (63%) | 0.006 |
| **Nerve Injury** |  |  |  |  |
| **Median nerve** | 135 (43%) | 31 (37%) | 104 (45%) | 0.27 |
| Different branch |  |  |  | 0.55 |
| Motor branch only | 34 (11%) | 7 (8%) | 27 (12%) |  |
| Sensory branch only | 31 (10%) | 6 (7%) | 25 (11) |  |
| Motor and Sensory | 70 (22%) | 18 (22%) | 52 (23%) |  |
| **Ulnar nerve** | 64 (20%) | 19 (23%) | 45 (20%) | 0.63 |
| Different branch |  |  |  | 0.69 |
| Motor branch only | 45 (14%) | 15 (18%) | 30 (13%) |  |
| Sensory branch only | 14 (5%) | 3 (4%) | 11 (5%) |  |
| Motor and Sensory | 5 (2%) | 1 (1%) | 4 (2%) |  |
| **Peroneal nerve** | 111 (36%) | 24 (29%) | 87 (38%) | 0.19 |
| **Tibial nerve** | 77 (25%) | 12 (15%) | 65 (28%) | 0.02 |
| **Sural nerve** | 56 (18%) | 9 (11%) | 47 (20%) | 0.07 |
| **Nerve injuries in multiple areas** | | | | 0.12 |
| Nerve injured in one area | 51 (16%) | 18 (22%) | 33 (14%) |  |
| Nerve injury in two areas | 73 (23%) | 18 (22%) | 55 (24%) |  |
| Nerve injury in three areas | 34 (11%) | 3 (4%) | 31 (14%) |  |
| Nerve injury in four areas | 34 (11%) | 8 (10%) | 26 (11%) |  |
| Nerve injury in five areas | 22 (7%) | 5 (6%) | 17 (7%) |  |
| Nerve injury in six areas | 11 (4%) | 1 (1%) | 10 (4%) |  |
| Nerve injury in seven areas | 6 (2%) | 2 (2%) | 4 (2%) |  |
| Nerve injury in eight areas | 1 (0%) | 0 (0%) | 1 (0%) |  |
| **Number of different types of nerve injuries*** | | | | 0.09 |
| One type of injury | 95 (30%) | 30 (36%) | 65 (28%) |  |
| Two types of injury | 81 (26%) | 15 (18%) | 66 (29%) |  |
| Three types of injury | 41 (13%) | 6 (7%) | 35 (15%) |  |
| Four types of injury | 12 (4%) | 3 (4%) | 9 (4%) |  |
| Five types of injury | 3 (1%) | 1 (1%) | 2 (1%) |  |
| **Pathological classification** |  |  |  | 0.17 |
| Demyelination only | 67 (21%) | 18 (22%) | 49 (21%) |  |
| Axonal loss only | 64 (20%) | 17 (21%) | 47 (20%) |  |
| Demyelination combined with axonal loss | 101 (32%) | 20 (24%) | 81 (35%) |  |
| **Peripheral neuropathy on different sides of the body** | | | | 0.24 |
| Left side only | 32 (10%) | 7 (8%) | 25 (11%) |  |
| Right side only | 23 (7%) | 7 (8%) | 16 (7%) |  |
| Both sides | 177 (57%) | 41 (49%) | 136 (59%) |  |
|  | **Total**  **（n=313）** | **6-month follow-up (n=83)** | **12-month follow-up (n=230)** | **p-value** |
| **Classification by anatomical location of motor branch** | | | |  |
| Proximal limb only | 0 (0%) | 0 (0%) | 0 (0%) | - |
| Distal limb only | 53 (17%) | 15 (18%) | 38 (17%) | 0.88 |
| Proximal and distal limbs | 164 (52%) | 38 (46%) | 126 (55%) | 0.20 |
| **F-wave abnormality** | 22 (7%) | 3 (4%) | 19 (8%) | 0.24 |
| **F-wave abnormality on different sides of the body** | | |  | 0.41 |
| Left side only | 12 (4%) | 1 (1%) | 11 (5%) |  |
| Right side only | 8 (3%) | 2 (2%) | 6 (3%) |  |
| Both sides | 2 (1%) | 0 (0%) | 2 (1%) |  |
| **Nerve with F-wave abnormality** | | | | 0.50 |
| Median nerve only | 19 (6%) | 3 (4%) | 16 (7%) |  |
| Ulnar nerve only | 2 (1%) | 0 (0%) | 2 (1%) |  |
| Median and ulnar nerve | 1 (0%) | 0 (0%) | 1 (0%) |  |

^*^ One type of nerve contains the same nerve on both sides of body
